# A45 ASSESSING THE ROLE OF MICROBIOTA DYSBIOSIS ON INTESTINAL TUFT CELL FUNCTIONS UPON *GIARDIA* INFECTION

**DOI:** 10.1093/jcag/gwad061.045

**Published:** 2024-02-14

**Authors:** O Sosnowski, T Allain, D McKay, A G Buret

**Affiliations:** University of Calgary, Calgary, AB, Canada; University of Calgary, Calgary, AB, Canada; University of Calgary, Calgary, AB, Canada; University of Calgary, Calgary, AB, Canada

## Abstract

**Background:**

Enteric tuft cells can detect and respond to intestinal parasitic infection by modulating host responses to aid parasite clearance, yet the extent of tuft cell functions upon other infections remains to be elucidated. Previous work in our lab showed that the enteric protozoan parasite *Giardia* induced tuft and goblet cell hyperplasia, yet *Giardia* colonization in *Pou2f3*^*-/-*^ (tuft cell-deficient) mice was impaired. Not only can *Giardia* modulate the gut microbiota and lead to microbial dysbiosis, but the microbiota itself can influence *Giardia* colonization. Interestingly, certain microbiota-derived products can activate tuft cells in the gut. In this study, we investigated the role of microbiota dysbiosis in the crosstalk between *Giardia* and tuft cells.

**Aims:**

We aim to explore the bidirectional effects between tuft cells and *Giardia* infection by investigating the role of 1) the host microbiota influenced by the presence or absence of tuft cells in altering *Giardia* infectivity, and 2) *Giardia* modified microbiota in contributing to tuft cell activity.

**Methods:**

Fecal matter from 6–8-week-old C57Bl/6 and *Pou2f3*^*-/-*^ mice were administered to polyethylene glycol (PEG) treated C57Bl/6 mice via oral gavage for 7 days, followed by gavage with 5x10^4^*Giardia muris* trophozoites. Duodenal parasite burden was assessed 11 days after infection. Small intestinal (SI) microbiota was collected from 5–7-week-old C57Bl/6 and *Pou2f3*^*-/-*^ mice following 11 days of *G. muris* infection and transplanted into PEG treated C57Bl/6 and *Pou2f3*^*-/-*^ recipients. 11 days post-transfer, SI tuft and goblet cell hyperplasia was assessed in recipient mice by immunohistochemistry, and *Dclk1, Atoh1,* and *Muc2* gene expression was quantified by RT-qPCR. Parameters of gut motility were evaluated.

**Results:**

FMT with *Pou2f3*^*-/-*^ mouse fecal matter prior to *Giardia* infection led to improved weight gain during infection compared to FMT from C57Bl/6 mice. *Giardia* modified microbiota (GMM) did not induce tuft or goblet cell hyperplasia 11 days after transfer. Jejunal *Dclk1, Atoh1*, and *Muc2* were no different after GMM compared to control microbiota (CM) transfer. Ileal *Atoh1* or *Muc2* were upregulated in mice receiving GMM from C57Bl/6 or *Pou2f3*^*-/-*^ mice, respectively. Intestinal transit time and stool water content was altered in mice which received GMM from *Pou2f3*^*-/-*^ mice compared to CM.

**Conclusions:**

The resident microbiota in tuft cell deficient mice may in part contribute to limiting *Giardia* infection. *Giardia* modified microbiota does not substantially contribute to tuft cell hyperplasia or related gene expression alterations at 11 days after microbiota transfer. The mechanistic contributors to tuft cell hyperplasia during *Giardia* infection and how a lack of tuft cells can lead to impaired colonization does not appear to be primarily driven by microbiota alterations and remains to be fully defined.

**Funding Agencies:**

NSERC

